# Quality and Reliability of YouTube Videos on Surgical Treatment of Uterine Leiomyomas

**DOI:** 10.7759/cureus.20044

**Published:** 2021-11-30

**Authors:** Aslihan Ergul

**Affiliations:** 1 Obstetrics and Gynecology, Istinye University, GOP Medical Park Hospital, Istanbul, TUR

**Keywords:** leiomyomas, youtube, mici, global quality score, discern

## Abstract

Introduction

To determine the quality of English language YouTube videos on uterine leiomyomas (UL) and their surgical treatment.

Methods

The present study was performed from October 1 to October 8, 2021. A gynecologist with 10 years of experience searched for keywords on YouTube, including ‘UL surgery’, ‘laparoscopic myomectomy,’ ‘myomectomy,’ ‘surgical treatments for UL,’ and ‘operations for UL.’ Videos were categorized into three groups according to content as informative videos, personal experience videos, and news update videos. All videos characteristics, including the number of views, the length and duration of the video, number of ‘likes,’ ‘dislikes,’ and ‘comments,’ were recorded. Medical information and content index (MICI) score, DISCERN score, and Global Quality Score (GQS) were calculated for each video.

Results

In total, 54 videos were categorized as informative videos, 46 videos were classified as patient experience videos, and 37 videos were accepted as news agency videos. The mean comment numbers were 105.6 for patient experience videos, and the difference was statistically different in favor of patient experience videos (p= 0.001). The GQS and DISCERN scores were significantly higher for the informative group in comparison with the other two groups (p=0.001 and p=0.001 for both groups). Clinical symptoms and treatment outcomes were the most frequently mentioned content in informative videos (81.8% and 97.1%). The mean MICI score was 2.7.

Conclusion

The present study demonstrated that YouTube videos about UL and its surgical treatments have low quality and utility. However, informative videos that are mostly uploaded by professional health providers have significantly better DISCERN and GQS scores.

## Introduction

Uterine leiomyomas (UL) are an extremely common gynecologic pathology of the uterus, and up to 77% of women of reproductive age are diagnosed with UL [1]. Previous reports proved the relationships between UL with dysmenorrhea, menorrhagia, pelvis pain, miscarriage, and infertility [2]. Numerous different treatment modalities, including drug therapy, embolization of UL, ablation with ultrasound, and open and laparoscopic myomectomy, were described for UL [3]. Technological advances facilitating communication, difficulties in reaching hospitals and doctors due to COVID-19, patient willingness to benefit from more than one source of information and get opinions from more than one source have increased the use of other information sources, including television, newspapers, and social media.

Online information resources, such as e-libraries, websites, and social media applications, are commonly preferred ways to obtain information in the present age. Additionally, Freeman and colleagues stated that sources with visual content attract more attention than audio texts and written sources [4]. Recently, YouTube, which was founded in 2005, has become the largest social media application, and billions of videos are uploaded to this platform [5]. Previous reports emphasized the public interest and significance of YouTube videos as information sources for patients, patient relatives, and professional healthcare providers. Kumar et al. analyzed YouTube videos about hypertension, and the authors stated that the videos with higher view rates were misleading [6]. In another study, Bora and colleagues stated that YouTube videos about infectious diseases had poor quality [7].

Although many studies investigated the quality of YouTube videos on gynecologic diseases, to our knowledge, no study evaluated the quality of YouTube videos on UL and its surgical management. In the present study, we tried to determine the quality of English language YouTube videos about UL and its surgical treatments.

## Materials and methods

The present study was performed from October 1 to October 8, 2021. A gynecologist with 10 years of experience searched for keywords on YouTube, including ‘UL surgery,’ ‘laparoscopic myomectomy,’ ‘myomectomy,’ ‘surgical treatments for UL,’ and ‘operations for UL.’ In total, 181 YouTube videos between two and 16 minutes in length were analyzed for inclusion in the study. Non-English YouTube videos, videos with content unrelated to UL surgery, and videos with personal ads were excluded from the study. Eventually, 137 videos met the study inclusion criteria, and all videos were recorded on a playlist for evaluation by an experienced gynecologist. Institutional Ethics Committee approval was not obtained due to no patient data being used.

Initially, videos were categorized into three groups according to content as informative videos (videos that mentioned accurate information and proven surgical methods for UL), personal experience videos (videos describing patients' experiences during UL diagnosis and surgery), and news update videos (videos about surgical options for UL and uploaded by new agencies). The number of views, length of the video, and duration of the video on YouTube were noted for each video. Additionally, the number of ‘likes,’ ‘dislikes,’ and ‘comments’ were recorded. Also, videos were classified according to the upload source as professional healthcare providers, non-professional people, and news agencies. Lastly, all videos were divided into two groups according to the target population as ‘healthcare professional’ and ‘patients and patient relatives.’

Medical information and content index (MICI) Score, DISCERN score, and global quality score (GQS)

The MICI questionnaire was developed for objective assessment of video content and the MICI form (range from zero point to five points, worst to best) was used to analyze the content of informative videos according to the absence or presence of five headings, including the prevalence of disease, transmission, possible symptoms, screening test, and alternative treatment options [8]. The DISCERN questionnaire includes five ‘yes’ or ‘no’ questions about video utility and quality, and a ‘yes’ answer equals one point and a ‘no’ answer means zero points. The DISCERN score is obtained by summing the scores for the questions [9]. The global quality score (GQS) was determined to analyze video quality, and each video was scored from worst to best, between one and five. A score of one means video with poor quality, most information missing, not at all useful for patients, and a score of five means excellent quality and very useful for patients [10]. The MICI questionnaire (supplementary file 1), DISCERN questionnaire (supplementary file 2), GQS questionnaire (supplementary file 3, and the details of the scoring criteria of the questionnaires are given in the Appendices.

Statistical analysis

Data analysis was done using the Statistical Package for the Social Sciences version 20 (SPSS IBM Corp., Armonk, NY, USA) program. Comparison of independent groups was performed using the t-test. The χ2 and Fisher exact tests were used for the comparison of categorical data. A post hoc test was used to compute pairwise comparisons. Quantitative data are shown as mean ± standard deviation values. Categorical data are presented as frequency (n) and percentages (%). The data were evaluated at the 95% confidence level and a p-value of less than 0.05 was accepted as statistically significant.

## Results

**Figure 1:**
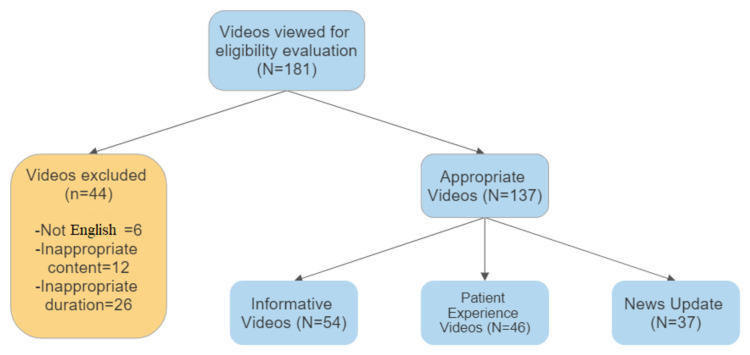
Flowchart for video selection

At the end of the evaluation, 181 videos were analyzed for inclusion in the study, and 44 videos were excluded from the study (six videos were in a language other than English, 12 videos had inappropriate content, and 26 videos had an inappropriate duration). In total, 54 videos were categorized as informative videos, 46 videos were classified as patient experience videos, and 37 videos were accepted as news agency videos (Figure 1).

The number of views, video length, duration of the video on YouTube, and likes and dislikes of videos were similar between informative videos, patient experience videos, and news agency videos (p=0.334, p=0.612, p=0.359, p=0.076, and p= 0.289, respectively). In contrast, mean comment numbers were 105.6 for patient experience videos, and the difference was statistically different in favor of patient experience videos (p= 0.001). The GQS was 3.7 for informative videos, 2.4 for patient experience videos, and 2.6 for news agency videos (p= 0.001). Similarly, the DISCERN score was significantly higher for informative videos (p=0.001). Informative videos were mostly uploaded by professional individuals and videos targeting professional health care providers were significantly common among informative videos (p=0.001 and p=0.035) (Table 1).

**Table 1:** Analysis of video features by category

Characteristics	Informative	Patient experience	News update	p-value
Number of videos	54	46	37	
Audience interaction parameters*				
Number of views	6184.5±4468.3	4736.1±3091.7	4696.5±2918.3	0.334
Video length (min)	5.6±3.6	5.9±3.9	6.1±3.2	0.612
Duration on YouTube (days)	246.4±176.2	245.3±177.8	317.1±267.2	0.359
Likes	49.9±42.3	52.0±42.4	63.3±41.2	0.076
Dislikes	11.2±8.6	10.3±6.6	12.9±9.7	0.289
Comments	17.6±14.0	105.6±58.6	25.1±19.2	0.001
Global quality score*	3.7±1.0	2.4±1.1	2.6±0.9	0.001
DISCERN score	2.7±0.9	1.6±0.9	1.7±0.9	0.001
Source of upload				0.001
Professional individuals	35	13	5	
Non-professional individuals	11	20	15	
News agencies	8	13	17	
Target audience				0.035
For doctors and healthcare providers	14	4	3	
For patients	40	42	34	

Comment numbers were significantly higher for patient experience videos in comparison to informative videos and news agency videos (p=0.001 and p=0.001). However, the GQS and DISCERN scores were significantly higher for the informative group in comparison with the other two groups (p=0.001 and p=0.001 for both groups). Pairwise comparison analysis of groups is summarized in Table 2.

**Table 2:** Pairwise comparisons of video groups according to usefulness Values of p<0.05 were accepted as significant and marked bold.

Characteristics	p-value		
	Informative vs Patient experience	Informative vs News update	Patient experience vs News update
Comments	0.001	0.137	0.001
Global quality score	0.001	0.001	0.806
DISCERN score	0.001	0.001	0.992
Source of upload	0.001	0.001	0.161
Target audience	0.036	0.053	0.987

Clinical symptoms and treatment outcomes were the most frequently mentioned content in informative videos (81.8% and 97.1%). The mean MICI score was 2.7 and MICI score components are presented in Table 3.

**Table 3:** Detailed content analysis of informative videos based on MICI scores * mean ± standard deviation MICI: medical information and content index

Component of MICI scale	Videos with information	MICI score*
Prevalence	56 (40.9%)	0.5±0.5
Transmission	15 (10.9%)	0.2±0.1
Clinical symptoms	112 (81.8%)	0.8±0.4
Screening/tests	90 (65.7%)	0.4±0.5
Treatment/outcomes	133 (97.1%)	0.8±0.5
Total MICI score		2.7±1.3

## Discussion

Easy access to information in the field of health on the internet has changed the way patients receive information about their diseases. Previous data revealed that almost 95% of individuals who use the internet watch YouTube videos [11]. Thus, we aimed to analyze YouTube videos about UL and its surgical management, one of the most common diseases in gynecology practice. We found that patient experience videos had a significantly higher comment rate. Additionally, informative videos had significantly better DISCERN and GQS scores and were mainly uploaded by professional health care providers.

The DISCERN score was described and externally validated for the assessment of video quality as an information source. Ferhatoglu and colleagues analyzed YouTube videos about obesity surgery and stated that videos shared by professional health care providers had a significantly better DISCERN score [12]. In another study that analyzed the quality of YouTube videos about coronavirus disease 2019 (COVID-19) and female sexual function, the authors claimed that YouTube videos uploaded by professional health care providers had a higher DISCERN score but stated YouTube videos had low quality [13]. Similarly, the GQS score was used to determine the quality of YouTube videos. Kılınc and Sayar used the GQS score to evaluate YouTube videos about orthodontics and emphasized that YouTube videos about orthodontics had low quality [14]. In the present study, we found that YouTube videos about UL surgical treatment were inadequate and of low quality. However, videos that were shared by professional health care providers had significantly higher DISCERN and GQS scores in comparison with patient experience videos and news agency videos.

The MICI score was created by Napgal and colleagues to evaluate the video quality in informative videos about the Ebola virus epidemic [15]. Since then, many authors used the MICI score to assess video quality in different disciplines. Sevgili and Baytaroglu analyzed informative videos about cardiologic diseases and COVID-19 and found that the MICI score was 4.1 [16]. In another study, Atac et al. investigated the quality of YouTube videos about COVID-19, and the authors stated that English videos had a score of 2.76 [17]. Most studies used the MICI score for infectious diseases, but our study is the first to use the MICI score to assess YouTube videos about UL and its surgical treatments. The MICI score for YouTube videos in our study was very low. We believe that the lack of transmission in UL, as in infectious diseases, and the low score for this question may have led to this result. New nomograms about chronic diseases will be better in the assessment of these videos.

YouTube videos with higher comment numbers receive more interaction. However, Yuksel and Cakmak did not find any correlation between comment numbers among informative videos, patient experience videos, and news agency videos [13]. Similarly, Sevgili and Baytaroglu did not find a significant difference with regard to comment number in their study, which investigated the quality of YouTube videos about coronary artery disease (16). In contrast, patient experience videos had significantly higher comment numbers in the present study. Patients trying to obtain information from healthcare professionals and patients with similar diseases may be a factor in this result.

The source that uploads the video to YouTube affects the accuracy and reliability of the video. In the Atac study that analyzed YouTube videos, the most useful videos were uploaded by news agencies [17]. In another study, Baytaroglu and Sevgli evaluated YouTube videos about peripheral vascular disease, and they did not find any correlation between the source of upload and the type of video [18]. In contrast, Yuksel and Cakmak found informative videos were shared mostly by professional healthcare providers [13]. In the present study, most informative videos were uploaded by professional health care providers.

The present study has some limitations. First, we performed the assessment only in English. However, English is the most commonly used language on YouTube. Second, in the present study, only five words were searched on YouTube, but the selected five words are the most chosen words on YouTube. Last, the study covered a certain period, but information about this subject is constantly updated.

## Conclusions

Our study demonstrated that YouTube videos about UL and its surgical treatments have low quality and utility. However, informative videos that ate mostly uploaded by professional health providers have significantly better DISCERN and GQS scores. We propose that uploading more informative videos by healthcare professionals will improve the quality of YouTube videos.

## Appendices

Supplementary file 1

**Table 4:** MICI scale to assess the quality of the content of videos Each item is awarded 1 point if mentioned in the video; a maximum score of 5 in each component. SARS-CoV-2: severe acute respiratory syndrome coronavirus 2; MICI: medical information and content index

Component	Description of scoring
Prevalence	Number of globally/locally confirmed cases reported
	Number of globally/locally suspected cases reported
	Number of global/local deaths reported
	Mentions about populations at high risk
	Number/proportion of patients who are severely ill
Transmission	Location of origin of the virus
	Human to human transmission (including spread via droplets)
	Mentions about spread from contact with contaminated surfaces
	Mentions about basic precautionary measures (wearing mask handwashing and social distancing)
	Mentions about the incubation period
Signs and Symptoms	Common symptoms: fever, tiredness, dry cough
	Other symptoms: shortness of breath, aches and pains, sore throat
	Less common symptoms: diarrhea, nausea, or a runny nose
	Emergency warning signs for COVID-19 that require medical attention immediately (like trouble breathing, persistent pain or pressure in the chest, new confusion or inability to arouse, bluish lips or face)
Screening/Testing	Mentions that some people become infected but do not develop any symptoms and don't feel unwell
	Mentions the test uses respiratory secretion to test
	Uses PCR to check and identify SARS-CoV-2
	Shows how the test is done
	Mentions criteria for testing/screening
Treatment/ Outcome	Mentions that mild symptoms can be self-resolving
	Mentions that some patients become more ill (mentions Hospitalization, ICU admission) and die
	Mentions that treatment is supportive
	Mentions that vaccines are in the making – none currently available
	Mentions about the rational use of medical masks

Supplementary file 2

**Table 5:** Modified DISCERN instrument 1 point per question answered yes

Modify Discern (1 point per question answered yes)
1. Is the video clear, concise, and understandable?
2. Are valid sources cited? (from valid studies, physiatrists, or rheumatologists)
3. Is the information provided balanced and unbiased?
4. Are additional sources of information listed for patient reference?
5. Does the video address areas of controversy/uncertainty?

Supplementary file 3

**Table 6:** Global Quality Scale

Score	Global score description
1	Poor quality, poor flow of the site, most information missing, not at all useful for patients
2	Generally poor quality and poor flow, some information listed but many important topics missing, of very limited use to patients
3	Moderate quality, suboptimal flow, some important information is adequately discussed but others poorly discussed, somewhat useful for patients
4	Good quality and generally good flow, most of the relevant information is listed, but some topics not covered, useful for patients
5	Excellent quality and excellent flow, very useful for patients
